# Morphine dependence in single enteric neurons from the mouse colon requires deletion of *β*‐arrestin2

**DOI:** 10.14814/phy2.12140

**Published:** 2014-09-04

**Authors:** Tricia H. Smith, Joy Ngwainmbi, Atsushi Hashimoto, William L. Dewey, Hamid I. Akbarali

**Affiliations:** 1 Department of Pharmacology and Toxicology, Virginia Commonwealth University, Richmond, Virginia, USA

**Keywords:** Colon, enteric neurons, morphine

## Abstract

Chronic administration of morphine results in the development of tolerance to the analgesic effects and to inhibition of upper gastrointestinal motility but not to colonic motility, resulting in persistent constipation. In this study we examined the effect of chronic morphine in myenteric neurons from the adult mouse colon. Similar to the ileum, distinct neuronal populations exhibiting afterhyperpolarization (AHP)‐positive and AHP‐negative neurons were identified in the colon. Acute morphine (3 *μ*M) decreased the number of action potentials, and increased the threshold for action potential generation indicative of reduced excitability in AHP‐positive neurons. In neurons from the ileum of mice that were rendered antinociceptive tolerant by morphine‐pellet implantation for 5 days, the opioid antagonist naloxone precipitated withdrawal as evidenced by increased neuronal excitability. Overnight incubation of ileum neurons with morphine also resulted in enhanced excitability to naloxone. Colonic neurons exposed to long‐term morphine, remained unresponsive to naloxone suggesting that precipitated withdrawal does not occur in colonic neurons. However, morphine‐treated colonic neurons from *β*‐arrestin2 knockout mice demonstrated increased excitability upon treatment with naloxone as assessed by change in rheobase, number of action potentials and input resistance. These data suggest that similar to the ileum, acute exposure to morphine in colonic neurons results in reduced excitability due to inhibition of sodium currents. However, unlike the ileum, dependence to chronic exposure of morphine develops in colonic neurons from the *β*‐arrestin2 knockout mice. These studies corroborate the in‐vivo findings of the differential role of neuronal *β*‐arrestin2 in the development of morphine tolerance/dependence in the ileum and colon.

## Abstract (precis)

Tolerance to chronic morphine does not develop in the colon, resulting in persistent constipation. Here, we examined electrophysiological characteristics of colonic neurons isolated from adult mouse and find that precipitated withdrawal occurs in neurons from the ileum but not colon. However, enhanced neuronal excitability in colonic neurons reflecting dependence to morphine occurs in colonic neurons of *β* ‐arrestin2 knockout mice. These studies show that mechanisms of morphine dependence–like tolerance differ between the ileum and colon and require downregulation of *β* ‐arrestin2.

## Introduction

Opioids are one of the most commonly prescribed drugs; over 90% of chronic pain patients utilize these medications. Extended opioid use leads to Opioid Bowel Syndrome with the primary clinical characteristics of nausea, bloating, vomiting, and constipation [for review see (Grunkemeier et al. 2007)]. Morphine acts on the *μ*‐opioid receptors located on enteric neurons, causing an acute decrease in neurotransmitter release (Schaumann 1955; Paton 1957), and a decrease in the firing rate of myenteric neurons (North and Tonini 1977). This correlates with reduced gastrointestinal motility, causing severe constipation (De Luca and Coupar 1996; Wood 2006). Enteric neurons display electrophysiological properties that fall into two major categories; afterhyperpolarization (AH) and synaptic (S) neurons (Clerc et al. 1998; Hirst et al. 1974; Rugiero et al. 2002) for review see (Nurgali 2009). Previous studies in the primary cultured adult mouse ileum neurons identified a population of afterhyperpolarization‐positive (AHP) and afterhyperpolarization‐negative (AHP‐negative) neurons (Smith et al. 2012). Morphine reduced the neuronal excitability of AHP‐positive neurons of the adult mouse ileum neurons but had no effect on AHP‐negative neurons (Smith et al. 2012). The reduction in excitability was characterized by an increase in the threshold at which an action potential (AP) fires, a decrease in the number of APs elicited, and a decrease in the AP amplitude. The amplitude of sodium currents were significantly reduced resulting from a shift to an inactivated state (Smith et al. 2012).

Repeated and prolonged morphine administration results in tolerance to the analgesic effects of morphine, but seemingly minimal tolerance develops to constipation (Ross et al. 2008). Isolated tissue studies in the organ bath show that tolerance does develop in the ileum but not the colon (Ross et al. 2008). Interestingly, the development of tolerance in the ileum was coupled with a decrease in the levels of *β*‐arrestin2 while neither a decrease in *β*‐arrestin2 levels nor tolerance developed in the colon (Kang et al. 2012). Previous studies have shown that acute morphine administration in *β*‐arrestin2 knockout mice abrogates morphine‐induced decrease in colonic transit (Raehal et al. 2005) suggesting that morphine signaling requires the presence of *β*‐arrestin2. Studies of the isolated tissues of *β*‐arrestin2 knockout ileum and colon demonstrate that repeated administration results in tolerance development in both the ileum and colon (Kang et al. 2012; Maguma et al. 2012).

In order to determine whether tolerance and/or dependence develops in isolated enteric neurons from the ileum and the colon, we determined the effects of long‐term morphine exposures followed by the *μ*‐opioid receptor antagonist naloxone on neuronal excitability. Similar to the ileum, AHP‐positive but not AHP‐negative neurons from the colon were sensitive to morphine, but unlike the ileum, response to precipitated withdrawal was not observed in the colon. However, precipitated withdrawal could be induced in colonic neurons from the *β*‐arrestin2 knockout mice. These studies lend support to the concept that differences in the ability of morphine to regulate *β*‐arrestin2 levels in the ileum and colon define the development of tolerance and dependence in these two tissues of the gastrointestinal tract.

## Methods

### Ethical approval

All animal care and experimental procedures were in accordance with and approved by the Institutional Animal Care and Use Committee at Virginia Commonwealth University.

### Isolation and culture of cells from adult mouse myenteric plexus

Myenteric neurons from the adult mouse ileum and colon were prepared as described recently (Smith et al. 2012, 2013). All chemicals and reagents were obtained from Sigma Aldrich (St Louis, MO), unless otherwise noted, except cell culture reagents, which were purchased from Gibco (Grand Island, NY). Male Swiss Webster mice (25–30 g; Harlan Sprague Dawley, Inc., Fredrick, MD, USA), C57/Bl6 or *β*‐arrestin2 KO mice (25–30 g,) were killed by cervical dislocation. The breeding pairs for the *β*‐arrestin2 KO mice were obtained from Dr. Lefkowitz (Duke University, Durham, NC) and housed within the transgenic facility at Virginia Commonwealth University. The animals were housed up to five per cage with access to food and water ad libitum. The mice were kept in the animal facility for 1 week to permit acclimation prior to the experiments. Every effort was made to reduce the use of animals to the minimum number required to achieve sufficient statistical power. Ileum and colon were immediately dissected and placed in ice‐cold Krebs solution (in mmol/L: 118 NaCl, 4.6 KCl, 1.3 NaH_2_PO_4_, 1.2 MgSO_4_ 25 NaHCO_3_, 11 glucose, and 2.5 CaCl_2_) bubbled with carbogen (95% O_2_/5% CO_2_). Segments of either colon or ileum were threaded longitudinally on a plastic rod through the lumen and the longitudinal muscle with the myenteric plexus (LMMP) was gently removed using a cotton‐tipped applicator. LMMP strips were rinsed three times in 1 mL Krebs and gathered by centrifugation (350 × *g*, 30 s). LMMP strips were then minced with scissors and digested in 1.3 mg/mL collagenase‐type II (Worthington) and 0.3 mg/mL bovine serum albumin in bubbled Krebs (37°C) for 1 h, followed by 0.05% trypsin for 7 min. Following each digestion, cells were triturated and collected by centrifuge (350 × *g* for 8 min). Cells were then plated on laminin (BD Biosciences, San Jose, CA, USA) and poly‐D‐lysine‐coated coverslips in Neurobasal A media containing B‐27 supplement, 1% fetal bovine serum, 10 ng/ml glial cell line‐derived neurotrophic factor (GDNF; Neuromics, Edina, MN), and antibiotic/antimycotic liquid. Half of the cell media was changed every 2–3 days.

Where indicated, isolated cells from the ileum and colon were incubated with 3 *μ*mol/L morphine. Following isolation, cells were allowed to establish in culture for 1–4 days in complete neuron media. In one series of experiments after the cells adhered to the plate, the media was replaced with that containing 3 *μ*mol/L morphine for 1–2 days before whole‐cell patch‐clamp experiments. Cells were maintained in morphine prior to addition of naloxone in the recording chamber.

### Immunocytochemistry

For immunohistochemistry, the isolated cells were cultured for 2 weeks on coverslips to allow for axonal growth and then fixed in 4% formaldehyde for 30 min. Cells were permeabilized with 0.01% Triton X‐100 in PBS (30 min), and blocked with 10% goat serum (1 h). Preparations were incubated with the primary antibody overnight at 4°C. Primary antibodies used were as follows: Neural‐specific anti‐*β*III‐tubulin (rabbit, Abcam ab18207‐100, 1:100 RRID:AB_444319), anti‐glial fibrillary acidic protein (GFAP, mouse, Chemicon MAB360, 1:500 RRID:AB_2109815).

Following three washes with PBS, cells were incubated with the appropriate secondary antibodies; goat anti‐rabbit Alexa 488 Dye (Molecular Probes, RRID:AB_143165 1:1,000, 1 h, RT) and goat anti‐mouse Alexa 594 Dye (Molecular Probes, RRID:AB_141593 1:1,000, 1 h, RT). Visualization was performed on an Olympus Fluoview Confocal Microscope and software (v5.0).

### Electrophysiology

Neuronal cells were studied after plating on coverslips. Cells were placed in an experimental chamber and perfused (1–2 mL/min) with an external physiological solution containing (in mmol/L): 135 NaCl, 5.4 KCl, 0.3 NaH_2_PO_4_, 5 HEPES, 1 MgCl_2_, 2 CaCl_2_, and 5 glucose. Patch electrodes (2–4 MΩ) were pulled from borosilicate glass capillaries (Sutter Instruments, CA) and filled with internal solution containing (in mmol/L): 100 K‐aspartic acid, 30 KCl, 4.5 ATP, 1 MgCl_2_, 10 HEPES, and 0.1 EGTA. Whole‐cell patch‐clamp recordings were made with an Axopatch 200B amplifier (Molecular Devices, Sunnyvale, CA) at room temperature and pulse generation and data acquisition were achieved with Clampex and Clampfit 10.2 software (Molecuar Devices). Initial cell characteristics were determined in current clamp mode with current provided in 13 sweeps of 0.5 s duration ranging from −0.03 to 0.09 nA in 0.01 nA increments. Current–voltage relationships were determined in voltage‐clamp mode, in 16,500 ms‐sweeps beginning at −100 mV and increasing in 10 mV intervals to 50 mV. Current amplitudes were normalized to cell capacitance (pF) to determine current density. Action potential (AP) derivatives were determined using the differential function in Clampfit software. The threshold of APs were determined as the voltage at which the d(V)/d(T) function deviated from zero. AP height was determined by measuring the threshold to the peak of the AP. Voltage‐dependence of steady‐state inactivation and activation were determined and fit via Boltzman distribution as described previously (Akbarali and Giles 1993).

### Surgical pellet implantation

Mice were anesthetized with 2.5% isoflurane. Hair was shaved on the dorsum at the base of the neck and the area cleaned with 10% providone iodine (General Medical, Prichard, WV) and then 70% ethanol. Adequate anesthesia was noted by the absence of the righting‐reflex, corneal reflex and lack of toe‐pinch response, according to IACUC guidelines. A 1‐cm transverse incision was made into the subcutaneous space toward the dorsal flank to insert a 75 mg morphine‐pellet (National Institute of Drug Abuse, Bethesda, MD) distal to the incision. The site was closed with sterile surgical autoclips (MikRon AutoClip 9‐mm wound clips, Becton Dickinson, MD) and iodine was applied to the surface. The animals were returned to their home cages for recovery and assessed daily for the next 72 h for signs of inflammation or infection at the surgical site; animals were euthanized if these signs were observed.

### The tail‐flick immersion test

To assess nociception, mice were gently held under a cloth by the experimenter and the distal 1/3 of the tail immersed in a water bath set at 56°C. The mouse flicked its tail out of the bath at the first sign of discomfort, and this time was counted as a baseline control tail‐flick time (2–4 s). Morphine (10 mg/kg, s.c.) was administered and after 20 min. a test tail‐flick time was obtained. A 10 s cutoff time was used to prevent tail damage. Antinociception was quantified as the percentage of maximum possible effect, calculated as: %MPE = [(test time−control time)/(10−control time)] × 100.

### Data analysis

Results are presented as mean ± SEM for the number of cells (*n*). Statistical tests were performed using GraphPad Prism 5.0 software using Two‐way ANOVA or two‐tailed *t*‐test. Values of *P* < 0.05 were regarded as significant.

## Results

### Immunohistochemistry of cultured cells derived from the adult mouse colon LMMP preparation

**Figure 1.**
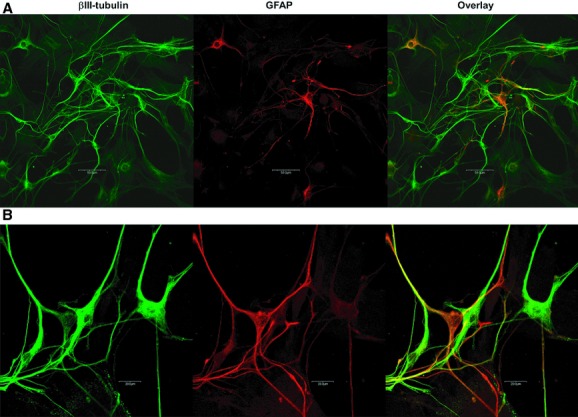
Neurons and glia cells from the colonic longitudinal muscle/myenteric plexus (LMMP) preparation. Confocal microscopy revealed neuronal‐specific β III‐tubulin (first column; Abcam rabbit, 1:1500) staining and staining by the glial marker GFAP (second column, Chemicon, mouse 1:500) in cells isolated from the LMMP preparation of the colon (A). (B) at higher magnification, the close proximity of neurons and glia is evident. Antibodies were visualized with appropriate goat secondary antibody Alexa 488 (green, Molecular probes, 1:1000) or Alexa 594 (red, Molecular Probes, 1:1000). No staining was seen when primary antibody was omitted (data not shown).

Isolated cells were derived from the longitudinal muscle myenteric plexus (LMMP) preparation of the adult mouse colon. Interestingly, we observed that fewer neurons could be isolated from the colon than the ileum. Cells attached to the poly‐d‐lysine‐ and laminin‐coated slips after 1 day in culture and long neural or glial projections were evident after 2 days. After a week in culture, cells began to form clusters which appeared ganglionic in nature. Staining was performed after 15 days in culture to allow maximal cell adhesion and cell projection growth. Mouse colonic neurons stained positively for the neuronal‐specific marker *β*III‐tubulin (Fig. 1, first column), while glia were visualized with glial‐specific marker GFAP (Fig. 1, second column). Neurons and glia grow in close proximity and may potentially interact in in vitro (overlay, C).

### Electrophysiological characteristics of colonic neurons from the adult mouse

Whole‐cell patch‐clamp recordings were carried out on neurons from 1 to 3 days in culture derived from the adult mouse colon (*n* = 18). Initial characterization utilized a current clamp protocol of 13 sweeps beginning with a current injection of −0.03 nA for 200 ms, and increasing stepwise by 0.01 to 0.09 nA with a 15 s start‐to‐start sweep interval. In voltage‐clamp protocol, the neurons were clamped at Vh = −60 mV and then clamped for 300 ms beginning at −100 mV and increasing to +50 mV in 10 mV steps.

**Figure 2.**
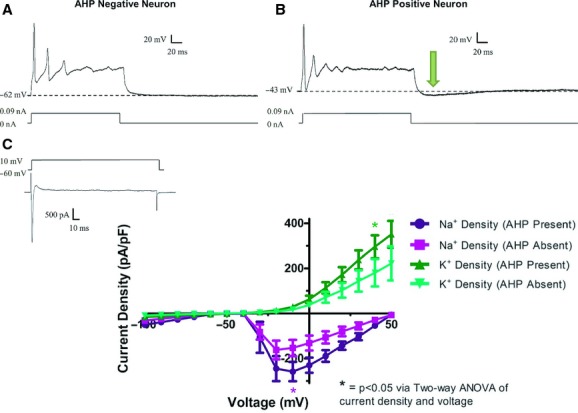
Cultured neurons from the adult mouse colon consist of two electrophysiologically distinct populations. In current clamp mode (A, B), all neurons exhibit action potentials upon current injection of 0.09 nA. At the end of current pulse, neurons either returned to their original resting membrane potential (A), or dipped below baseline in a slow after‐hyperpolarization (AHP, arrow, B). AHPs have an average magnitude of −7.06 ± 1.09 mV, an initial duration of 198.23 ± 14.8 ms, and a *τ* = 109.7 ± 29.2. In voltage‐clamp mode (C), inward Na^+^ currents follow by a sustained outward K^+^ current are readily apparent (insert). Current density–voltage relationships of Na^+^ and K^+^ currents in both AHP‐negative and AHP‐positive neurons showed that AHP‐positive neurons had significantly greater current densities as determined by two‐way ANOVA (*P > 0.05).

Neurons were identified by their *sine que non*, or defining feature, the action potential (AP; Fig. 2A,B). Additionally, neurons were characterized by the presence (*n* = 9) or absence (*n* = 9) of an afterhyperpolarization (AHP, Fig. 2B). The average magnitude of the AHP was −7.06 ± 1.09 mV and a length of 198.2 ± 14.8 ms. The AHP *τ* = 109.7 ± 29.2 ms.

Both neuron subtypes fired either single or multiple action potentials. Both subtypes also largely fired in a “phasic” manner at maximum current injection (0.09 nA, Table 1; 7 AHP positive neurons and 6 AHP‐negative neurons) which consisted of multiple action potentials followed by a plateau. The AP properties were not different between AHP‐positive and negative neurons. There was no difference in the threshold (current) or voltage at which an AP was elicited, the duration of the action potential at 30% of its height, or the height of the action potential.

**Table 1.** Electrophysiological characteristics of enteric neurons from the adult mouse colon.

	AHP‐positive neurons	AHP‐negative neurons
Cell capacitance (pF)	10.9 ± 2.4	11.5 ± 2.6
Resting membrane potential (mV)	−47.6 ± 2.0	−60.4 ± 2.3*
Threshold (nA)	0.015 ± 0.001	0.022 ± 0.005
Action potential initiation (mV)	−13.06 ± 3.01	−17.88 ± 2.97
Action potential duration (at 30% height, ms)	2.43 ± 0.11	2.31 ± 0.17
Action potential height (mV)	74.41 ± 6.56	84.36 ± 6.17
Input resistance (MΩ)	998.4 ± 406.6	608.9 ± 230.2
Firing of action potentials (Phasic/Tonic)	(7/2)	(6/3)

* P <0.5; t‐test.

Neurons that lacked an AHP had a more polarized resting membrane potential (RMP) than AHP‐positive neurons (−60.4 ± 2.3 mV in AHP‐negative, neurons vs. −47.6 ± 2.0 in AHP‐positive neurons; *t*‐test, *P* < 0.05). However, there was no difference in the passive properties of these two cell types, including cell capacitance, and input resistance.

In voltage‐clamp experiments, the magnitude of peak inward and outward currents was significantly larger in AHP‐positive neurons compared to AHP‐negative neurons. A representative voltage‐clamp tracing (Fig. 2C, insert) shows that a neuron depolarized to +10 mV from −60 mV displayed a sharp inward current followed by a sustained outward current. AHP‐positive neurons had significantly larger peak inward current densities (in pA/pF: 258.2 ± 43.0 in AHP‐positive vs. −153.4 ± 14.9 in AHP‐negative) and larger outward sustained current densities (in pA/pF: 350.8 ± 50.9 in AHP‐positive vs. 221.9 ± 28.7 in AHP‐negative) as compared to AHP‐negative neurons (*P* < 0.05, determined by two‐way ANOVA of voltage and current density).

### Effect of acute morphine on neuronal excitability

**Figure 3.**
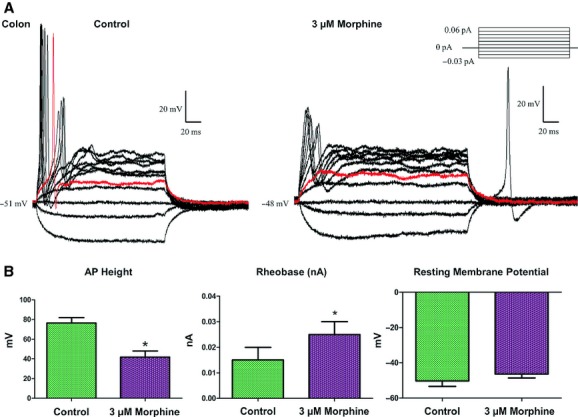
Morphine decreases neuronal excitability in AHP‐positive neurons of the adult mouse colon without changing the resting membrane potential. In current clamp mode (A), colonic neurons fire multiple action potentials in the absence of drug (left panel). The red trace refers to the rheobase at which action potentials are first elicited. Following 3 *μ*mol/L morphine treatment (right panel), action potentials are noticeably blunted, only single action potentials fire, and the neuron no longer fires at the previous rheobase. Statistical analysis (B) shows a significant increase in AP height, and a significant increase in rheobase without an alteration in the resting membrane potential of the neuron, determined by paired *t*‐test, significance at *P* < 0.05.

Morphine (3 *μ*mol/L) significantly attenuated neuronal excitability in AHP‐positive neurons (*n* = 5). In current clamp mode (Fig. 3A), a representative trace of an AHP‐positive neuron showed that morphine significantly decreased the height of the action potential (76.34 ± 5.78 mV before morphine, 41.82 ± 5.25 mV after morphine), increased the rheobase (0.015 ± 0.001 nA before morphine to 0.025 ± 0.005 nA after morphine), and inhibited the neuron from firing multiple action potentials. The RMP was not affected by morphine. Similar to our previous finding in ileum neurons (Smith et al. 2012), AHP‐negative neurons did not respond to morphine (data not shown).

**Figure 4.**
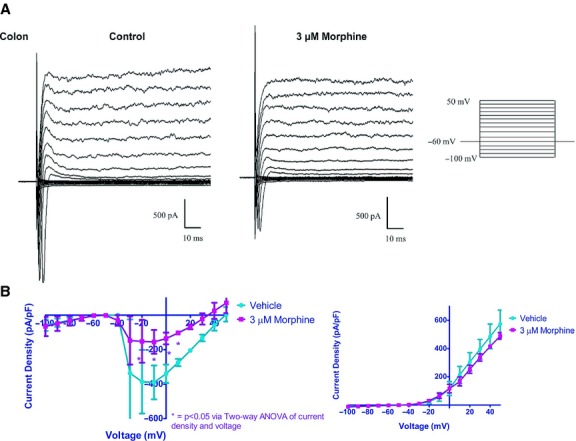
Morphine decreases inward Na^+^ current density without affecting large sustained outward K^+^ current density. In voltage‐clamp mode (A), net currents were measured for duration of 300 ms demonstrating inward Na^+^ and outward K^+^ currents. Morphine (3 *μ*mol/L) significantly reduced peak inward currents but not outward currents. (B) I–V curves for peak inward and end of pulse outward currents in the absence and presence of morphine. Two‐way ANOVA of current density and voltage (*P < 0.05).

In voltage‐clamp experiments, morphine (3 *μ*mol/L) reduced the inward current density of colonic neurons but not the sustained outward K^+^ currents (Fig. 4), similar to the effects previously observed on ileum neurons (Smith et al. 2012).

### Effect of chronic morphine

Tolerance to the effects of morphine in 5‐day morphine‐pelleted mice was determined by the warm‐water tail withdrawal (*n* = 10). Morphine‐pelleted mice achieved an %MPE value of 10.8% ± 3.5%, consistent with previous reports (Smith et al. 2002) indicative of tolerance. Enteric neurons were isolated from antinociceptive tolerant mice.

**Figure 5.**
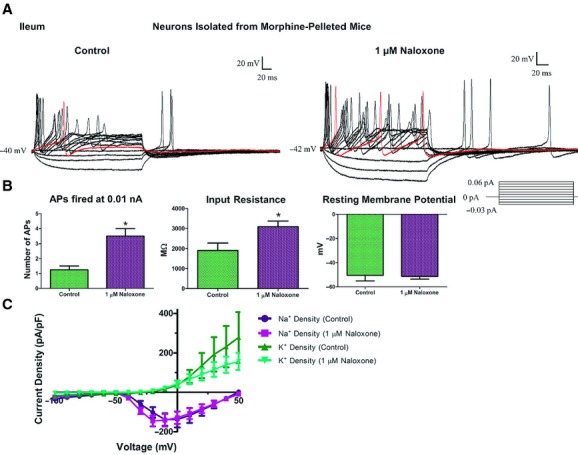
Precipitated withdrawal caused hyper excitability in morphine‐tolerant AHP‐positive enteric neurons from the ileum. Enteric neurons were isolated from 5‐day morphine‐pelleted mice and maintained in cell culture containing 3 *μ*mol/L morphine. In current clamp mode (A), the neuron from tolerant mouse ileum (left panel) fires robust action potentials at 0.01 nA (red line). Following 1 *μ*mol/L naloxone treatment (right panel), significantly greater number of action potentials were elicited at the rheobase. (B) Bar graph illustrating number of action potentials at 0.01 nA, the input resistance and the resting potential in the absence and presence of naloxone from morphine‐tolerant mouse ileum. (C) Current–voltage relationship for peak inward Na^+^ and outward K^+^ currents in the absence and presence of naloxone. Two‐way ANOVA of voltage and current density, significance at *P* < 0.05.

Current clamp experiments revealed that ileal neurons isolated from tolerant animals and maintained in 3 *μ*mol/L morphine cell media (Fig. 5A, left) behave similarly to untreated neurons (Figs. 2A,B, 3A; and Smith et al. 2012); the tolerant neurons fire robust, multiple action potentials at a low rheobase (0.012 ± 0.002 nA). Upon administration of naloxone (1 *μ*mol/L, Fig. 5A, right, *n* = 6), the number of action potentials fired at 0.01 nA significantly increased from 1.2 ± 0.2 APs to 3.5 ± 0.5 APs (*P* < 0.05, paired *t*‐test, Fig. 5B). This increase in AP firing rate was paired with an increase in input resistance from 1710 ± 349 MΩ to 2672 ± 285 MΩ. The threshold at which the action potential was initiated (mV), the height of the action potential, and the duration of the action potential (ms) at 30% of its height was not affected. The RMP of the neuron did not change. Furthermore, the overall peak inward and sustained outward current densities were not altered when examined in voltage clamp in the absence and presence of naloxone (Fig. 5C).

**Figure 6.**
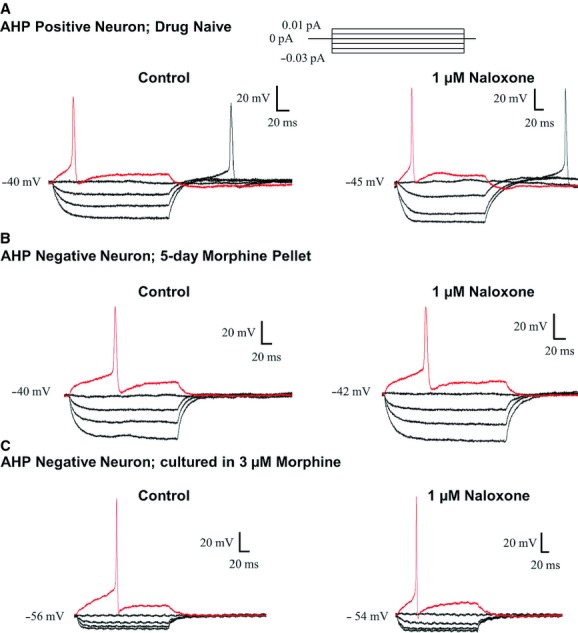
Precipitated withdrawal does not occur in the absence of morphine tolerance, or in AHP‐negative neurons. Raw traces demonstrating lack of enhanced cell excitability in response to naloxone from (A) AHP‐positive neuron that was not pretreated with morphine, (B) an AHP‐negative neuron obtained from ileum of morphine‐pelleted mouse, and (C) an AHP‐negative neuron from the ileum that was pretreated with morphine (overnight).

Naloxone (1 *μ*mol/L) did not affect AHP‐positive neurons from drug naïve mice, or the AHP‐negative neurons isolated from morphine‐pelleted mice or AHP‐negative neurons incubated with morphine overnight (Fig. 6).

The above data indicate that enhanced excitability occurs following naloxone treatment in ileal neurons exposed to long‐term morphine and rendered antinociceptive tolerant. We were unable to isolate sufficient viable neurons from the colon of morphine‐pelleted mice. Gross observation of these animals showed a profound effect of chronic morphine administration on the colon. The colon appeared more vascularized and inflamed, and stomach contents and the amount of fecal material were drastically increased, consistent with the presence of constipation. Colon‐derived neurons did not survive in culture, and thus appear to be more sensitive to overt damage by long‐term morphine administration. Further experiments were therefore conducted in which colon neurons were isolated into regular complete media and then administered morphine overnight after 1 day in culture.

**Figure 7.**
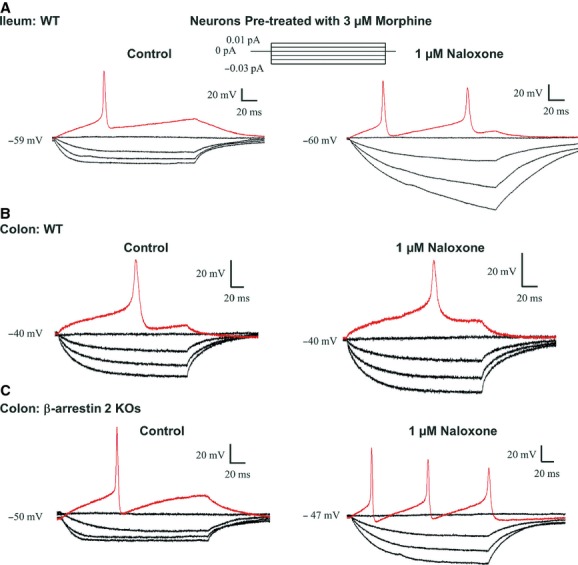
Genetic ablation of *β*‐arrestin two leads to the development of morphine dependence in AHP‐positive enteric neurons from the colon. Raw traces from enteric neurons preincubated with morphine for 48 h. (A) Ileum neuron in the presence of naloxone fired multiple action potentials at rheobase and demonstrated increased input resistance. (B) AHP‐positive colon neuron showed on increase in number of action potentials or increased input resistance in the presence of naloxone. (C) Colon neuron from *β*‐arrestin2 knockout mice responded to naloxone with an increased number of action potentials and enhanced input resistance.

We first tested whether precipitated withdrawal manifested in ileal neurons after overnight incubation with 3 *μ*mol/L morphine (Fig. 7A, *n* = 6). Similar to ileal neurons from morphine‐pelleted mice, overnight incubation (16 h) rendered mice in opioid withdrawal as evidenced by the effect of naloxone. The number of action potentials and input resistance significantly increased. Precipitated withdrawal was not seen in neurons derived from the colon of wild‐type mice. Colonic neurons incubated with 3 *μ*mol/L morphine overnight did not respond to naloxone (*n* = 5) (Fig. 7B). However, precipitated withdrawal did occur in colonic neurons isolated from *β*‐arrestin 2 knockout mice (Fig. 7C). The number of action potentials fired at 0.01 nA significantly increased from 0.60 ± 0.24 APs before naloxone to 2.00 ± 0.57 APs following 1 *μ*mol/L naloxone (*n* = 6). The rheobase reduced from 0.030 ± 0.010 nA (control) to 0.020 ± 0.006 nA following naloxone. Additionally, the input resistance increased from 3330 ± 898 MΩ (control) to 3900 ± 905 MΩ (naloxone) (paired *t*‐test, *P* < 0.05).

## Discussion

Enhanced excitability of enteric neurons from the ileum upon precipitated withdrawal has been examined extensively in isolated tissue segments since the early studies of Collier and colleagues (Collier et al. 1981). In the present study we have extended these findings to determine the effects of naloxone‐induced withdrawal in individual isolated neurons from the ileum and colon of the adult mouse and demonstrate that while precipitated withdrawal occurs in the AHP‐positive neurons of the ileum, it does not manifest in drug naïve or AHP‐negative neurons. Furthermore, precipitated withdrawal does not occur in chronically morphine‐treated colonic neurons. However, withdrawal responses are observed in colonic neurons from *β*‐arrestin2 knockout mice. Withdrawal responses are a hallmark of dependence therefore these results suggest that dependence occurs in the ileum but not the colon following chronic morphine treatment. This is similar to our previous studies in which tolerance to repeated morphine develops in the isolated segments of ileum but not the colon. However, tolerance (Kang et al. 2012) and dependence (this study) occur in the isolated neurons from the colon of *β*‐arrestin2 knockout mice. While the cellular basis for tolerance and dependence are likely to differ, the finding that in both cases *β*‐arrestin2 is required to prevent these effects in the colon suggests common pathways. Further studies will be required to delineate the two components, that is, tolerance and dependence in colonic neurons.

The findings reported in this study characterize two major electrophysiological classes of enteric neurons in the adult mouse colon and delineate the acute and chronic effects of morphine on these cells. Previously, we used whole‐cell patch‐clamp recordings to characterize the enteric neurons of the adult mouse ileum (Smith et al. 2012). The present study offers the first whole‐cell electrophysiological assessment of neurons isolated from the adult mouse colon. Enteric neurons from both the ileum and the colon can be divided into two major classes, those with and without an afterhyperpolarization (AHP). As found in the ileum, colonic neurons that exhibit an AHP have significantly larger peak inward currents and sustained outward currents. The RMP of ileal and colonic AHP‐positive neurons were not different as are the action potential (AP) characteristics. However, the action potentials were initiated at a less polarized threshold in the colon compared to the ileum (−13.06 ± 3.01 mV in the colon vs. −26.4 ± 2.4 mV in the ileum) for AHP‐positive neurons, suggesting that colonic neurons are less excitable than their AHP‐positive counterparts in the ileum. In general, the isolation of neurons from the colon was technically more difficult with less neurons/isolation. The specific reason for this is not clear at present but may reflect differences in the coupling of the myenteric plexus within the longitudinal versus the circular muscle in the two tissues(Furukawa et al. 1986). Microelectrode recordings have established that morphine decreases neuronal firing rates (North and Tonini 1977; Karras and North 1981). In ileum AHP‐positive enteric neurons, morphine decreases neuronal excitability by attenuating sodium currents by driving sodium channels into an inactivated state (Smith et al. 2012). The same mechanism of action appears to be responsible for decreased neuronal excitability in the colon. As in the ileum, morphine administration to the colon caused a reduction in AP number, a decrease in AP height, and an increased threshold at which an AP fires. This is accompanied by a marked decrease in peak inward sodium current. (Smith et al. 2012). These results differ from findings in the guinea pig, which attribute the reduction in excitability to hyperpolarization of the enteric neurons (North 1976; North and Tonini 1977).

In the present study, 5‐day morphine‐pellet implantation in the mouse significantly increases AHP‐positive neuronal excitability of neurons from the ileum following naloxone administration, denoted by a significant increase in AP firing rates and an increase in the input resistance of the cell. The increase in input resistance upon precipitated withdrawal by an opioid antagonist suggests channel closures may be responsible for the increased excitability, such as the closure of an inwardly rectifying potassium channel. Interestingly, we did not observe changes in the resting membrane of cells from either ileum or colon exposed to chronic morphine or following naloxone. Naloxone had no effects on AHP‐negative neurons from tolerant mice or in neurons from drug naive AHP‐positive neurons of the colon or ileum (Smith et al. 2012). The ionic basis for the increased input resistance requires further study.

*β*‐arrestin 2 was initially discovered as a large 418 amino acid protein responsible for the homologous desensitization of the *β*‐adrenergic receptors (Lohse et al. 1990, 1992). Indeed, *β*‐arrestin 2 binds to and ultimately internalizes many phosphorylated heterotrimeric GTP‐binding protein (G‐protein) coupled receptors (GPCRs), including the *μ*OR(Bohn et al. 1999). However, recent research has largely expanded our understanding of the role of *β*‐arrestin 2 beyond GPCR internalization to be a critical component in specific signaling cascades, including two mitogen‐activated protein kinase cascades (MAPK cascades); the extracellular‐signal‐regulated kinase (ERK) cascade (DeFea et al. 2000a,b; Luttrell et al. 2001) and the c‐jun n‐terminal kinase 3 (JNK3) cascade (McDonald et al. 2000; for review see (Pierce and Lefkowitz 2001). Interestingly, in the mouse gastrointestinal tract, *β*‐arrestin2 was found to be localized in cholinergic and *μ*‐OR‐expressing enteric neurons, but not in NOS or Substance P containing neurons. Additionally, a greater number of *β*‐arrestin2‐expressing neuronal cell bodies were observed in the ileum than in the colon (Maguma et al. 2014).

In summary, we have described the characteristics of cultured colonic neurons from the adult mouse. Furthermore, we have shown that *β*‐arrestin2 plays a dynamic role in the regulation of opioid dependence in the colon. The expression of *β*‐arrestin 2 prohibits tolerance and dependence in colonic enteric neurons.

## Conflict of Interest

All authors acknowledge no competing interest.
